# Uncertainty-Based Vibration/Gyro Composite Planetary Terrain Mapping

**DOI:** 10.3390/s19122681

**Published:** 2019-06-13

**Authors:** Chengchao Bai, Jifeng Guo

**Affiliations:** School of Astronautics, Harbin Institute of Technology, Harbin 150001, China; guojifeng@hit.edu.cn

**Keywords:** planetary rover, terrain mapping, vibration/gyro, measurement uncertainty, motion uncertainty, field test

## Abstract

Accurate perception of the detected terrain is a precondition for the planetary rover to perform its own mission. However, terrain measurement based on vision and LIDAR is subject to environmental changes such as strong illumination and dust storms. In this paper, considering the influence of uncertainty in the detection process, a vibration/gyro coupled terrain estimation method based on multipoint ranging information is proposed. The terrain update model is derived by analyzing the measurement uncertainty and motion uncertainty. Combined with Clearpath Jackal unmanned vehicle—the terrain mapping accuracy test based on ROS (Robot Operating System) simulation environment—indoor Optitrack auxiliary environment and outdoor soil environment was completed. The results show that the proposed algorithm has high reconstruction ability for a given scale terrain. The reconstruction accuracy in the above test environments is within 1 cm, 2 cm, and 6 cm, respectively.

## 1. Introduction

The planetary rover has different design requirements and configuration standards according to different task types and functions. As a key part of several subsystems, the navigation system provides the rover device with the ability to sense the environment. By carrying external sensing module (e.g., vision, lidar, etc.), the rover can acquire the structural features of the surrounding environment. Besides, the internal sensing module (e.g., inertial measurement unit, odometer, etc.) can be used to obtain the rover’s relative positional relationship with the environment. Lastly, through the fusion of multiple sensing modes, the rover’s global perception of the environment can be established. This is also well validated and applied in practical tasks such as the Spirit, Opportunity, etc. [1,2].

As the detection task becomes more complex, the detection time is longer, and the corresponding detection distance is also extended. This requires the rover itself to have accurate localization and environment reconstruction capabilities, providing accurate input to the planning system, which can optimize tour path to avoid the danger of obstacles and maximize the value of the mission. How to obtain high-precision and large-scale terrain sensing information in an unknown, a complex and dynamic environment, like planetary, is particularly important, which greatly affects the success or failure of the rover mission, and thus has become a research hot spot in this field [3,4,5].

The research on terrain perception can be analyzed from three aspects, namely sensor selection, algorithm and application [6]. First of all, in terms of sensor selection, the sensors used from the early stage are vision and lidar, and gradually developed to multisensor fusion. In 2007, Olson [7] achieved high-precision interframe matching in the vicinity of the rover by beam adjustment based on a large number of images taken during the in-orbit and rover process, and realized long-distance terrain reconstruction using wide baseline binocular vision, which purposed a possible solution to the realization of Mars long distance rover navigation. In 2011, Barfoot’s [8] team of the University of Toronto applied SLAM (Simultaneous Localization and Mapping) technology to planetary exploration, and achieved accurate global terrain estimation through laser radar and odometer compensation. The sparse feature method and batch alignment algorithm purposed in this research effectively solved the robustness problems of feature association and measurement outlier. Carrio et al. [9] proposed a SLAM terrain fusion estimation method based on a visual odometer, IMU, and wheel odometer. The innovation of the method is that it reconstructed the terrain through multisensor fusion, and improves the estimation accuracy by predicting the non-systematic error caused by the wheel interaction with odometer error model based on Gaussian process. Shaukat et al. [10] also proposed a fusion strategy of vision and lidar, and verified the advantages of this model in terms of distance, flexibility, and precision.

Secondly, in terms of the development of algorithms, the algorithm gradually evolved from the previous triangulation and filtering optimization to machine learning and biological inspiration. Li et al. [11] purposed a topological terrain estimate of the Spirit near Gusev Crater based on beam correction and generated a digital elevation map based on Ortho Maps. In 2013, the MRPTA (Micro-Rover Platform with Tooling Arm) project initiated by the Canadian Space Agency [12] realized the minimum sensor configuration-based terrain construction, proposed a local grid representation centered on the rover and used the map manager achieve the fusion management of measurements at different times. All of these works helped to achieve a globally optimized terrain representation. Later, Bajpai et al. [13] from Surrey University proposed a monocular-based planetary simultaneous localization and mapping technology (PM-SLAM). The innovation of this method was that it was inspired by the detection of biological semantic features and proposed visually significant model. First, this model gave a method for generating mixed marked features based on the point description, and then used the features estimate terrain state. The test results of multiple scenarios showed that the method could effectively improve the robustness of visual perception. At the same time, with the continuous development of deep learning and convolutional neural networks, more and more algorithms are applied to the rover mission [14].

Finally, in terms of application changes, the application gradually evolved from the initial terrain estimation to geographical environment modeling and scientific attribute detection. The overall trend is still more inclined to multisensor fusion terrain estimation that does not rely on the external environment condition. In 2017, Gonzalez et al. [15], from MIT, purposed a machine learning-based sliding detection method in the state of relying only on the internal sensing module to solve the sliding problem that rovers may encounter during the planetary exploration process. On one hand, since this method still used the original internal sensing device, the complexity of the system does not increase. On the other hand, it improves the adaptability to lighting conditions and compared and analyzed the detection validity of the supervised and unsupervised learning methods in the actual verification process. Recently, the research on the estimation of the surface structure of the planetary have made some achievements, and gradually extended to the next stage, namely multiattribute status terrain estimation. This new form of estimation is not only concerned with whether the ground flat or not, but also pay attention to fuse feature attributes such as hardness and material, etc. Deng’s [16] team, from the Harbin Institute of Technology, proposed a new idea of using the equivalent stiffness to characterize the pressure characteristics of the terrain and the friction angle to characterize the shear characteristics. They also proved that the interaction mechanics model between the wheel and the soil and the contact model for calculating the force between the wheel and the rock is equivalent, and purposed a digital elevation map with physical properties.

In addition, the terrain reconstruction problem is also a research hot spot in the field of ground robots and unmanned driving. As early as 2002, Professor Sebastian Thrun from Carnegie Mellon University gave a literature overview based on robotic drawing, and compared and analyzed a variety of different probability-based implementation methods. This work serves as a very representative work in the field, providing follow-up technical development support [17]. Bresson et al. [18] gave a literature overview of the development of SLAM technology and analyzed the current development trend of autonomous driving. Ye and Borenstein [19], from the University of Michigan, used 2D LIDAR to estimate the elevation map and certainty map of the terrain, and proposed the certainty assisted spatial filter, which effectively distinguish corrupted pixels in elevation maps through physical constraints in motion and spatial continuity. After that, Wolf [20] and others combined the terrain reconstruction and classification, and purposed three-dimensional terrain estimation based on the hidden Markov model. Besides, they distinguished the navigable area and the non-navigable area. Their work provides more in-depth information for later path planning and semantic ability in a certain sense. Gingras et al. [21] performed unstructured surface reconstruction using a 360-degree perspective lidar. By analyzing the surface and extracting the navigable space, the safely passable region is represented with compressed irregular triangular grid. This kind of compact terrain representation simplifies the computational complexity and ensures the reliability of the platform operation.

Although the above-mentioned sensing mode and terrain reconstruction method have achieved good precision and the effect is remarkable in practical applications, it is still necessary to consider how to maintain the ability to perceive the terrain under accidents (failure or partial failure) in uncertain external environments. Of course, there are many factors that may cause this kind of accidents, probably because the vibration frequency of the soft landing process is too large or the environmental condition, such as light and temperature, changes. Therefore, how to construct a more robust terrain perception capability based on existing terrain reconstruction capability is the next difficult point to be solved.

Based on the above analysis, this paper mainly focuses on the situation that the visual sensing unit cannot work normally when illumination condition changes. Besides, it discusses the feasible method of accurate terrain reconstruction by considering the active ranging information with motion uncertainty. As early as 1989, Hebert et al. [22] first proposed the use of the locus algorithm to construct terrain representations in spherical polar coordinate space. At the same time, effective registration of different detection position data was realized based on feature matching and iconic matching. On the basis of the former, Krotkov [23] considered the problem of target shadow occlusion. Then, in 2001, Whitaker [24] constructed a height function representation of the terrain based on multiple dense distance maps, and gave an optimal terrain estimate by looking for the maximum posteriori probability on the test set and the prior data set. Besides, his work verified that the results of multiple distance maps experiments are much better than any single distance maps. Finally, it is proved to be robust to noise with laser range finder. After that, Whitaker and Gregor [25] used the multi-viewpoint distance information with noise to estimate the surface configuration and gave the likelihood expression of the sensor model to construct the three-dimensional surface. Also, by optimizing the likelihood, an unbiased estimator was obtained. This method purposed new terrain estimation ideas compared with previous height measurements and recent point matching. Cremean and Murry [26] from Caltech proposed a 2.5D digital elevation map construction method suitable for high-speed and highly unstructured outdoor environments. First, they presented a complete uncertainty analysis of the distance measurement sensor error. Then, they transformed measurement into the measured probability density function by uncertain model, and selected the update region near the median region of the probability density function. Finally, the terrain result is continuously updated with Kalman filter, which purposes new ideas in the terms of previous local terrain estimation, update methods, as well as online implementations compared with former researches. Lshigami et al. [27] proposed a new fan-shaped reference grid for distance data transformation process to terrain, which helped to obtain an elevation map with cylindrical coordinates. This transformation method realizes the scaled representation of terrain representation, that is, to refine reconstruction near the rover, in which the farther away from the rover is, the more sparse the expression. Based on the previous research basis, Fankhauser [28], from ETH, proposed a probabilistic terrain estimation method for quadruped robots under uncertain localization conditions, and also considered the influence of sensor measurement error and platform motion estimation error. This method achieves high-precision terrain estimation based on kinematics and inertial measurement. At the same time, a three-dimensional covariance representation of the terrain was proposed and the map update error transfer relationship compared with former researches was also derived. Combined with the planetary rover environment, this paper mainly focuses on discussing the multipoint distance-based vibration/gyroscope-coupled elevation terrain construction method, so as to improve the robustness of rover to environmental changes and to provide support for subsequent motion planning and 3D-aware semantic field construction. The remainder of this paper is organized as follows.

Section 2 focuses on the terrain reconstruction method based on uncertainty analysis. Section 3 analyzes the depth distance sensor system and noise error used in the verification process. Section 4 compares and analyzes the rationality and correctness of algorithms based on ROS simulation platform and actual test environment. Finally, the conclusion is presented in Section 5.

## 2. Uncertainty-Based Terrain Mapping

### 2.1. Coordinate System Definition

**Figure 1:**
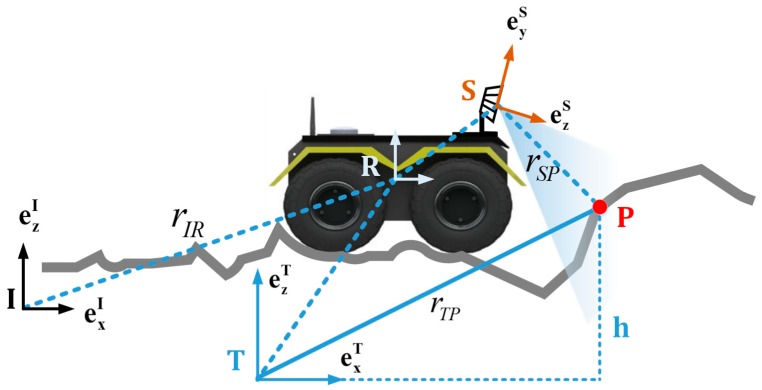
Coordinate system definition and conversion diagram.

This paper defines four coordinate systems: the inertial coordinate system I, the terrain coordinate system T, the rover body coordinate system R, and the sensor coordinate system S. The inertial coordinate system is fixed in the inertia space, the rover body coordinate system is fixed at the centroid position of the rover, and the sensor coordinate system is fixed with the centroid of the sensor body, as shown in Figure 1. The transformation relationship between the coordinate systems is given by the transformation matrix, that is, the three-dimensional translation r and three-dimensional rotation *ϕ*. The sensor and the rover are both fixedly calibrated when performing the task, so the relationship between the rover body coordinate system and the sensor coordinate system is known, and is defined here as $\left(r_{RS},\phi_{RS}\right)$. Similarly, the conversion between the inertial coordinate system and the rover body coordinate system is $\left(r_{IR},\phi_{IR}\right)$. Since the pose of the rover related to inertial coordinate system at different moments is highly uncertain, the covariance matrix of the pose at each moment is given synchronously:

(1) $$\sum_{IR}^{6\times 6}=Cov(r_{IR},\phi_{IR})$$

When the rover is advancing in the unknown terrain, the three-dimensional attitude can be described by pitch, yaw, and roll. This paper assumes that the terrain is fixed relative to the inertial coordinate system. The conversion between rover body and inertia coordinate system can be described by the following formula.

(2) $$\phi_{IR}=\phi_{I\tilde{R}}(\psi )\circ \phi_{\tilde{R}R}(\theta ,\varphi )$$

where $\phi_{I\tilde{R}}(\psi )$ describes that the inertial coordinate system rotates around the Z-axis ψ and turns into intermediate coordinate system $\tilde{R}$; $\phi_{\tilde{R}R}(\theta ,\varphi )$ describes that the pitch and roll transformations from the intermediate coordinate system to the rover system.

For subsequent derivation simplification and calculation, the inertial coordinate system is set to the Z-axis perpendicular to the ground and the Z-axis of the terrain coordinate system is always parallel to the Z-axis of the inertial coordinate system, that is, there is only one degree of freedom for the conversion between the two coordinate systems, which is also yaw around the Z-axis. Also, $\psi_{\left(I\to \tilde{R}\right)}$ is set to be given by $\psi_{\left(I\to T\right)}$, which means that the conversion of the terrain coordinate system and the rover body coordinate system has only two degrees of freedom of pitch and roll, thereby achieving dimensionality reduction between coordinate transformations.

### 2.2. Distance Information Associated with Terrain

For each measurement, there will be different numbers of sampling results according to the sensing ability of the sensing unit and the task requirements. To simplify the understanding, take a point here for analysis. As shown in Figure 1, point P is the measuring point, and its coordinates are $\left(x_{p},y_{p},{\tilde{h}}_{p}\right)$, which means that in the terrain coordinate system the height estimate at the point $\left(x_{p},y_{p}\right)$ is ${\tilde{h}}_{p}$. For the estimation of height, this paper uses Gaussian probability distribution to approximate it, which is ${\tilde{h}}_{p}\sim N(h_{p},\sigma_{h_{p}}^{2})$, where $h_{p}$ is the mean of the distribution and $\sigma_{h_{p}}^{2}$ is the variance of the distribution. As can be seen from Figure 1, the measured value of point P in the sensor coordinate system is rSPS, through the conversion of the sensor coordinate system to the terrain coordinate system we can get the formula

(3) hp=H[ΦST−1(rSPS)−rSMS)]

where H=[0 0 1]; the three-dimensional coordinates of the point P are extracted in the height direction. Furthermore, it can be known that the height estimation is directly related to the conversion matrix and the sensor measurement value, and corresponds to the error source of the previous analysis. Therefore, when we perform first derivative of the above equation, the Jacobian matrix corresponding to the error is obtained:

The sensor measuring Jacobian matrix:

(4) JS=∂hp∂rSPS=HC(ΦST)T

The sensor coordinate system rotation Jacobian matrix:

(5) JΦ=∂hp∂ΦST=HC(ΦST)TrSP×S

where C(Φ) is defined as a mapping corresponding to the rotation matrix, as detailed in the literature [29], i.e., $C:SO(3)\to {\mathbb{R}}^{3\times 3}$, Φ(r)≜C(Φ)r. Bringing the Jacobian matrix into the following equation, we can obtain the variance $\sigma_{h_{p}}^{2}$ error transmission:

(6) $$\sigma_{h_{p}}^{2}=J_{S}\sum_{S}J_{S}^{T}+J_{\Phi }\sum_{\Phi_{IS}}J_{\Phi }^{T}$$

The first item is sensor noise due to its error transmission, which is determined by the nature of the sensor itself. The covariance value is solved by the noise model, which is detailed shown in Section 3 for sensor model analysis. The second term is the error transmission caused by the conversion between coordinate systems. It should be noted that the conversion consists of two parts: translation and rotation. The definition of the terrain coordinate system is defined in the previous coordinate system definition, so the effect of translation can be ignored here. 

At this point, the noise error estimation based on the sensor measurement has been obtained, and for each measurement update there will be a corresponding height estimation so the next step can fuse the newly obtained height measurement estimate with the existing elevation terrain map. Because the height measurement estimate has no complex dynamic relationship with each measurement point, the state transfer equation is more intuitive, which is only measurement update for a certain point (x,y), so every point in the terrain map will be updated under each sensor measurement. On the contrary, if there is no update, the measurement will remain the same. A fusion form based on Kalman filtering is given here.

First, a simplified discrete Kalman filter equation is given:

Time update:

(7) $$\begin{array}{l} x_{k}=Ax_{k-1}+Bu_{k-1}\\ P_{k}=AP_{k-1}A^{T}+Q \end{array}$$

Status update:

(8) $$K_{k}=P_{k-1}H^{T}{\left(HP_{k-1}H^{T}+R\right)}^{-1}$$

(9) $$x_{k}=x_{k-1}+K_{k}(z_{k}-Hx_{k-1})$$

(10) $$P_{k}=(I-K_{k}H)P_{k-1}$$

For the rover terrain estimation, the state vector is actually the height scale of each measurement point, so H item is I, status $z_{k}$ corresponds to the current measurement point height estimate $h_{p}$, observation covariance R corresponds to the height estimate variance of the current measurement point $\sigma_{h_{p}}^{2}$, the observation value $x_{k}$ corresponds to the existing height value $\hat{h}$, and error covariance P corresponds to $\sigma_{h}^{2}$; substituting Equations (8)–(10) the following can be obtained.

(11) Kk=σh2(k−1)(σh2(k−1)+σhp2)−1

(12) h^(k)=h^(k−1)+Kk(hp−h^(k−1))

(13) σh2(k)=(I−Kk)σh2(k−1)

Substituting Equation (11) into Equation (12), the following is obtained.

(14) h^(k)=h^(k−1)+[σh2(k−1)(σh2(k−1)+σhp2)−1](hp−h^(k−1))=h^(k−1)+σh2(k−1)σh2(k−1)+σhp2(hp−h^(k−1))=h^(k−1)(σh2(k−1)+σhp2)+σh2(k−1)(hp−h^(k−1))σh2(k−1)+σhp2=h^(k−1)σhp2+σh2(k−1)hpσh2(k−1)+σhp2

Then, substituting Equation (11) into Equation (13) the following is obtained.

(15) σh2(k)=(I−Kk)σh2(k−1)=[I−(σh2(k−1)(σh2(k−1)+σhp2)−1)]σh2(k−1)=(I−σh2(k−1)σh2(k−1)+σhp2)σh2(k−1)=σh2(k−1)+σhp2−σh2(k−1)σh2(k−1)+σhp2σh2(k−1)=σhp2σh2(k−1)σh2(k−1)+σhp2

Thus the fusion of newly measured height $\left(h_{p},\sigma_{h_{p}}^{2}\right)$ with existing elevation map estimate $\left(\hat{h},\sigma_{h}^{2}\right)$ can be given, where *k* − 1 on the upper left represents the estimate before the update and *k* represents the updated estimate.

### 2.3. Motion Information Associated with Terrain

**Figure 2:**
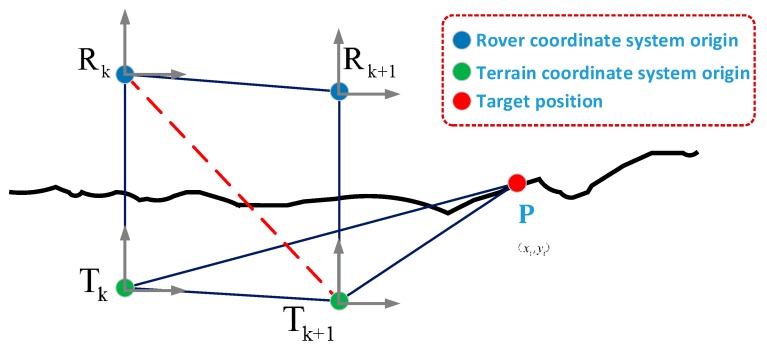
Positional relationship at different times.

In addition to the noise impact of the sensor itself, the motion of the rover will also produce noise errors. Unlike the conventional terrain estimation, this paper associates the terrain coordinate system with the motion ontology rather than the inertial coordinate system, so there will be terrain updates as long as the rover moves. As you can see from the previous section, in general, the mean and variance of each point will be updated according to the uncertainty of the motion, but this will bring huge computational pressure. So this section uses spatial covariance matrix of each point in real terrain to extend the structure of the elevation map, in this way the three-dimensional uncertainty information of each point can be obtained. According to the previous definition, the terrain coordinate system is associated with the current rover pose, in general, assuming there are new measurement updates with a point i in the grid, set its covariance as [30]

(16) $$\sum p_{i}=\left[\begin{array}{ccc} \sigma_{x,\min}^{2} & 0 & 0\\ 0 & \sigma_{y,\min}^{2} & 0\\ 0 & 0 & \sigma_{h}^{2} \end{array}\right]$$

where the height estimation variance $\sigma_{h}^{2}$ is given by the calculation in the previous section, the values of $\sigma_{x,\min}^{2}$ and $\sigma_{y,\min}^{2}$ approximate the uncertainty of the horizontal direction of the reaction, and its calculation is given by

(17) $$\sigma_{x,\min}^{2}=\sigma_{y,\min}^{2}={\left(d/2\right)}^{2}$$

where d is the length of the side of the square grid. Therefore, even if the sensor measurement update is not received at the current time, due to the relative motion change of the time before and after the rover, $\sum p_{i}$ will also be updated, which ensures the system’s robustness to motion noise. The terrain association derivation based on motion information is given below. It can be seen from Figure 2 that the representation of $r_{T_{k+1}P}$ in the terrain coordinate system at time k is as shown in the following equation.

(18) $$\begin{array}{ll} r_{T_{k+1}P} & =r_{T_{k+1}T_{k}}+r_{T_{k}P}\\ & =(r_{R_{k}T_{k}}-r_{R_{k}T_{k+1}})+r_{T_{k}P}\\ & =[r_{R_{k}T_{k}}-(r_{R_{k}R_{k+1}}+r_{R_{k+1}T_{k+1}})]+r_{T_{k}P}\\ & =-r_{R_{k}R_{k+1}}-r_{R_{k+1}T_{k+1}}+r_{R_{k}T_{k}}+r_{T_{k}P} \end{array}$$

Convert it to the *k* + 1 moment in terrain coordinate system, then

(19) rTk+1PTk+1=−ΦRk+1Tk+1−1(rRkRk+1Rk+1)−(rRk+1Tk+1Tk+1)+ΦTkTk+1−1(rRkTkTk+rTkPTk)

In this way, the value of measurement point in the terrain coordinate system can be given at each moment, but this also means that the newly estimated result at each moment is integrated with the previous existing result, which will also bring errors and complexity to the calculations. If it can ensure that terrain coordinate system at time *k* and *k* + 1 moment are consistent, the impact is avoided, and therefore the terrain map data does not need to be added or deleted, which is convenient for actual operation. Therefore, it is possible to make assumptions from translation and rotation, i.e.,

(20) $$r_{T_{k}T_{k+1}}=0$$

(21) $$\Phi_{T_{k}T_{k+1}}^{-1}=I$$

It can be seen from the above formula that the terrain coordinate system at time *k* and *k* + 1 is the same reference coordinate system, and further development of Equation (20) is available.

(22) $$r_{T_{k}T_{k+1}}=-r_{R_{k}T_{k}}+r_{R_{k}R_{k+1}}+r_{R_{k+1}T_{k+1}}=0$$

Unify it to the terrain coordinate system at time *k* + 1, then

(23) rRk+1Tk+1Tk+1=−ΦRk+1Tk+1−1(rRkRk+1)+ΦTkTk+1−1(rRkTkTk)

In the same way, the expansion of formula (21) is obtained.

(24) $$\Phi_{T_{k}T_{k+1}}^{-1}=\Phi_{R_{k}T_{k}}^{-1}\circ \Phi_{R_{k}R_{k+1}}\circ \Phi_{R_{k+1}T_{k+1}}=I$$

Unify it to the terrain coordinate system at time *k* + 1, then

(25) $$\Phi_{R_{k+1}T_{k+1}}={\left[\Phi_{R_{k}T_{k}}^{-1}\circ \Phi_{R_{k}R_{k+1}}\right]}^{-1}=\Phi_{R_{k}R_{k+1}}^{-1}\circ \Phi_{R_{k}T_{k}}$$

Therefore, combining Equations (19), (23), and (25) can lead to the following conclusions.

(26) rTk+1PTk+1=ΦTkTk+1−1(rTkPTk)

That is, the time *k* coordinate and the time *k* + 1 terrain coordinate system are aligned. The value of the point P in the terrain coordinate system at time *k* is equal to the value in the terrain coordinate system at time *k* + 1. For the dynamic process, the terrain representation reference is unified, which simplifies the difficulty of mass data fusion registrations.

Combined with Equation (19), the covariance transfer relationship due to motion at different times can be obtained.

(27) $$r_{T_{k+1}p}\sim N({\tilde{r}}_{T_{k+1}p},\sum_{P,k})$$

(28) $$\sum_{P,k+1}=J_{P}\sum_{P,k}J_{P}^{T}+J_{r}\sum_{P,k}^{r}J_{r}^{T}+J_{\Phi }\sum_{P,k}^{\Phi }J_{\Phi }^{T}$$

It can be known that there is correlation between the distance estimate $r_{T_{k+1}p}$ of point P at time *k* + 1 and the distance estimation $r_{T_{k}p}$ of point P at time *k* and the rover body coordinate transformation $\left(r_{R_{k}R_{k+1}},\Phi_{R_{k}R_{k+1}}^{-1}\right)$ from time *k* to *k* + 1, so the first derivative of Equation (19) can be used to obtain the corresponding Jacobian matrix.

1. Error transmission of time *k* + 1 caused by observation at time *k*.
  (29) JP=∂rTk+1PTk+1∂rTkPTk=C(ΦTkTk+1)T
  Combined with Equation (21), the following is obtained.
  (30) $$J_{P}=I$$
2. Error transmission caused by translation transformation of the rover body coordinate system from time *k* to time *k* + 1.
  (31) Jr=∂rTk+1PTk+1∂rRkRk+1Rk+1=−C(ΦRk+1Tk+1)T
3. Error transmission caused by rotation transformation of the rover body coordinate system from time *k* to time *k* + 1.
  (32) JΦ=∂rTk+1PTk+1∂ΦRkRk+1=∂[−ΦRk+1Tk+1−1(rRkRk+1Rk+1)−(rRk+1Tk+1Tk+1)+ΦTkTk+1−1(rRkTkTk+rTkPTk)]∂ΦRkRk+1=∂[−(ΦRkTk−1∘ΦRkRk+1)(rRkRk+1Rk+1)−(rRk+1Tk+1Tk+1)+(rRkTkTk+rTkPTk)]∂ΦRkRk+1=∂[−(ΦRkTk−1∘ΦRkRk+1)(rRkRk+1Rk+1)+ℵ]∂ΦRkRk+1=∂[−(ΦRkTk−1∘ΦRkRk+1)(ΦRkRk+1(rRkPRk)+(rRk+1PRk+1))+ℵ]∂ΦRkRk+1=−C(ΦRkTk)T(rRkPTk)×=−C(ΦRkTk)T(rRkTkTk+rTkPTk)×
  where ℵ=−(rRk+1Tk+1Tk+1)+(rRkTkTk+rTkPTk). So far, it is not enough to solve the covariance at time *k* + 1, but it also need to know the uncertainty influence caused by the motion estimation error from *k* to *k* + 1, i.e., solution of $\sum_{r}$, $\sum_{\Phi }$, which is expressed as follows.
  (33) $$r_{R_{k}R_{k+1}}\sim N({\tilde{r}}_{R_{k}R_{k+1}},\sum_{P,k}^{r})$$
  (34) $$\Phi_{R_{k}R_{k+1}}\sim N({\tilde{\Phi }}_{R_{k}R_{k+1}},\sum_{P,k}^{\Phi })$$

According to the previously defined coordinate system relationship, the Z-axis of the rover body coordinate system is aligned with the Z-axis of the inertial coordinate system and the obtained processed attitude uncertainty is only related to the yaw angle, so the covariance matrix representation of the pose of the rover at time *k* can be obtained through dimensionality reduction, i.e.,

(35) $$x_{IR}=(r_{IR},\Phi_{IR})\to x_{I\tilde{R}}=(r_{IR},\psi )$$

where, $\tilde{R}$ is the aligned rover body coordinate system, both satisfy equation $r_{IR}=r_{I\tilde{R}}$, $\psi =\psi_{I\tilde{R}}=\psi_{IR}$.

### 2.4. Covariance Solving Based on 3D Vibration/Gyro Detection

The Gaussian random model is used to approximate the motion process. At the same time, the external sensing unit is not used to realize the rover localization in this paper. Therefore, the localization mode using the triaxial vibration haptic sensing unit and the gyro is given. The following derivation method of $\sum_{P,k}^{r}$, $\sum_{P,k}^{\Phi }$ will be based on this mode.

#### 2.4.1. Solving Position Information Based on Vibration and Gyroscope Information

The experiment uses a three-axis vibration tactile sensor output value as the amplitude and frequency of the measurement point, which can be converted into an acceleration signal, that is, the input $a_{\tilde{R}}$; the single-axis gyroscope output is the angular acceleration around the Z-axis, which is recorded as $\beta_{\tilde{R}}$. In order to reduce the error caused by nonlinearity, the sensor connection point is taken as the coordinate origin and the interval before and after measurement Δt is taken. So there is

(36) $$\Delta v=\hat{a}\Delta t$$

where $\hat{a}=C_{I\tilde{R}}(\psi )a_{\tilde{R}}$ And, since the rover is relatively stable and slow during the course of travel, its displacement changes from time *k* to time *k* + 1 is obtained by

(37) Δx=ΔvΔt+12a^Δt2=a^Δt2+12a^Δt2≈32CIR˜(ψ)(aR˜R˜)Δt2

Similarly, based on the gyroscope input, the yaw angle change from *k* to *k* + 1 can be obtained, i.e.,

(38) $$\Delta \omega =C_{I\tilde{R}}(\psi )\beta_{\tilde{R}}\Delta t$$

(39) Δψ=ΔωΔt+12CIR˜(βR˜R˜)Δt2=CIR˜(ψ)(βR˜R˜)Δt+12CIR˜(ψ)(βR˜R˜)Δt2≈32CIR˜(ψ)(βR˜R˜)Δt2

#### 2.4.2. The Position Relationship between Time *k* and Time *k* + 1

In the actual sampling process, since the exist of uncertainty, the Gaussian noise vector is assumed to be $n={\left(n_{a},n_{\beta }\right)}^{T}$, therefore

(40) $$x_{I\tilde{R}}^{k+1}=x_{I\tilde{R}}^{k}+{\left[\Delta x\;\Delta \psi \right]}^{T}$$

Substituting Equations (37) and (39) into (40), we have

(41) [rIR˜k+1Iψk+1]=[rIR˜kIψk]+[32CIR˜(ψ)0032CIR˜(ψ)][ak+nakβk+nβk]Δt2

Performing the first derivative of the above equation and the Jacobian matrix of state and noise is given.

(42) $$J_{x}=\frac{\partial x_{I\tilde{R}}^{k+1}}{\partial x_{I\tilde{R}}^{k}}=\left[\begin{array}{cc} \frac{\partial r_{I\tilde{R}}^{k+1}}{\partial r_{I\tilde{R}}^{k}} & \frac{\partial r_{I\tilde{R}}^{k+1}}{\partial \psi_{I\tilde{R}}^{k}}\\ \frac{\partial \psi_{I\tilde{R}}^{k+1}}{\partial r_{I\tilde{R}}^{k}} & \frac{\partial \psi_{I\tilde{R}}^{k+1}}{\partial \psi_{I\tilde{R}}^{k}} \end{array}\right]=\left[\begin{array}{cc} I^{3\times 3} & \frac{3}{2}C_{I\tilde{R}}(\psi )(a_{k}+n_{a}^{k})\Delta t^{2}\\ 0^{1\times 3} & \frac{3}{2}C_{I\tilde{R}}(\psi )(\beta_{k}+n_{\beta }^{k})\Delta t^{2} \end{array}\right]$$

(43) $$J_{n}=\frac{\partial x_{I\tilde{R}}^{k+1}}{\partial n}=\left[\begin{array}{cc} \frac{\partial r_{I\tilde{R}}^{k+1}}{\partial n_{a}^{k}} & \frac{\partial r_{I\tilde{R}}^{k+1}}{\partial n_{\beta }^{k}}\\ \frac{\partial \psi_{I\tilde{R}}^{k+1}}{\partial n_{a}^{k}} & \frac{\partial \psi_{I\tilde{R}}^{k+1}}{\partial n_{\beta }^{k}} \end{array}\right]=\left[\begin{array}{cc} \frac{3}{2}C_{I\tilde{R}}(\psi )\Delta t^{2} & 0^{3\times 1}\\ 0^{1\times 3} & \frac{3}{2}C_{I\tilde{R}}(\psi )\Delta t^{2} \end{array}\right]$$

Then its covariance transfer relationship can be written as

(44) $$\sum_{I\tilde{R}}^{k+1}=J_{x}\sum_{I\tilde{R}}^{k}J_{x}{}^{T}+J_{n}\Omega J_{n}{}^{T}$$

In turn, the conversion relationship of the rover device from time *k* to time *k* + 1 can be obtained, that is,

(45) xR˜kR˜k+1=[rR˜kR˜k+1R˜k+1ψR˜kR˜k+1]

At the same time, the displacement and rotation angle values are decomposed.

(46) rR˜kR˜k+1R˜k+1=CIR(ψ)(rIR˜k+1−rIR˜k)

(47) $$\psi_{{\tilde{R}}_{k}{\tilde{R}}_{k+1}}=C_{IR}(\psi )(\psi_{I\tilde{R}}^{k+1}-\psi_{I\tilde{R}}^{k+1})$$

Substituting into Equation (45), the following equation is obtained.

(48) $$x_{{\tilde{R}}_{k}{\tilde{R}}_{k+1}}=\left[\begin{array}{c} C_{IR}(\psi )(r_{I\tilde{R}}^{k+1}-r_{I\tilde{R}}^{k})\\ C_{IR}(\psi )(\psi_{I\tilde{R}}^{k+1}-\psi_{I\tilde{R}}^{k+1}) \end{array}\right]=\frac{3}{2}\left[\begin{array}{c} a_{k}+n_{a}^{k}\\ \beta_{k}+n_{\beta }^{k} \end{array}\right]\Delta t^{2}$$

Then, the covariance of $x_{{\tilde{R}}_{k}{\tilde{R}}_{k+1}}$ can be obtained.

(49) $$\sum_{{\tilde{R}}_{k}{\tilde{R}}_{k+1}}=Cov(x_{{\tilde{R}}_{k}{\tilde{R}}_{k+1}})=\Omega {\left(\frac{3}{2}\Delta t^{2}\right)}^{2}$$

As can be seen from Equation (44),

(50) $$\Omega =J_{n}^{-1}(\sum_{I\tilde{R}}^{k+1}-J_{x}\sum_{I\tilde{R}}^{k}J_{x}{}^{T}){\left(J_{n}^{T}\right)}^{-1}$$

Substituting into Equation (49), then

(51) $$\sum_{{\tilde{R}}_{k}{\tilde{R}}_{k+1}}={\tilde{J}}_{n}(\sum_{I\tilde{R}}^{k+1}-{\tilde{J}}_{x}\sum_{I\tilde{R}}^{k}{\tilde{J}}_{x}{}^{T}){\tilde{J}}_{n}^{T}{\left(\frac{3}{2}\Delta t^{2}\right)}^{2}$$

where

(52) $${\tilde{J}}_{n}=J_{n}^{-1}={\left[\begin{array}{cc} \frac{3}{2}C_{I\tilde{R}}(\psi )\Delta t^{2} & 0^{3\times 1}\\ 0^{1\times 3} & \frac{3}{2}C_{I\tilde{R}}(\psi )\Delta t^{2} \end{array}\right]}^{-1}=\left[\begin{array}{cc} \frac{1}{\frac{3}{2}C_{I\tilde{R}}(\psi )\Delta t^{2}} & 0^{3\times 1}\\ 0^{1\times 3} & \frac{1}{\frac{3}{2}C_{I\tilde{R}}(\psi )\Delta t^{2}} \end{array}\right]$$

(53) $${\tilde{J}}_{x}\approx \left[\begin{array}{cc} I^{3\times 3} & C_{I\tilde{R}}(\psi )(r_{I\tilde{R}}^{k+1}-r_{I\tilde{R}}^{k})\\ 0^{1\times 3} & C_{I\tilde{R}}(\psi )(\psi_{I\tilde{R}}^{k+1}-\psi_{I\tilde{R}}^{k}) \end{array}\right]$$

The transition of the rover from *k* to *k* + 1 is determined by translation and rotation, so the covariance can be given by

(54) $$\sum_{{\tilde{R}}_{k}{\tilde{R}}_{k+1}}=\left[\begin{array}{cc} \sum_{P,k}^{r} & 0\\ 0 & \sigma_{\psi_{{\tilde{R}}_{k}{\tilde{R}}_{k+1}}}^{2} \end{array}\right]$$

Therefore, combined with Equations (51) and (54), the value of $\sum_{r}$ can be obtained. The last one that needs to be solved is $\sum_{P,k}^{\Phi }$. Since by defining coordinate system, this paper has this conclusion that only the orientation is changed, and the first derivative only stores the value of the z-axis, so it can be obtained:

(55) $$\sum_{P,k}^{\Phi }=J_{\psi }\sigma_{\psi_{{\tilde{R}}_{k}{\tilde{R}}_{k+1}}}^{2}J_{\psi }^{T}$$

In summary, combined with Equations (14)–(16) and (28), the uncertain terrain measurement updates of point P corresponds to every point in the terrain i from time *k* to time *k* + 1 can be obtained.

## 3. Sensor Noise Error Analysis

**Figure 3:**
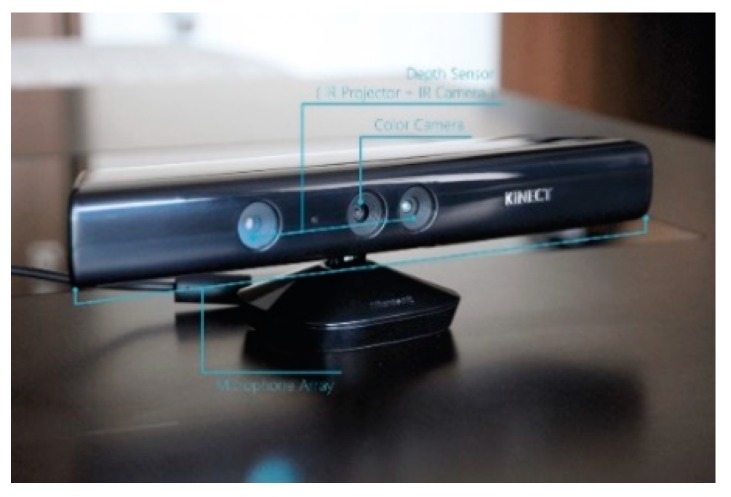
Microsoft Kinect V1.0 composition diagram.

This study uses Microsoft Kinect V1.0 as the depth sensor unit for verification. This sensor module is a depth camera sensor introduced by Microsoft in 2010 to detect environmental information by actively projecting structured light. Its composition is shown in the Figure 3, which consists of infrared projection, infrared camera, color camera, and microphone array. Some key parameters are given here, as shown in Table 1.

Based on the above parameter values, the system error and noise error are analyzed below.

### 3.1. Systematic Error Test

**Figure 4:**
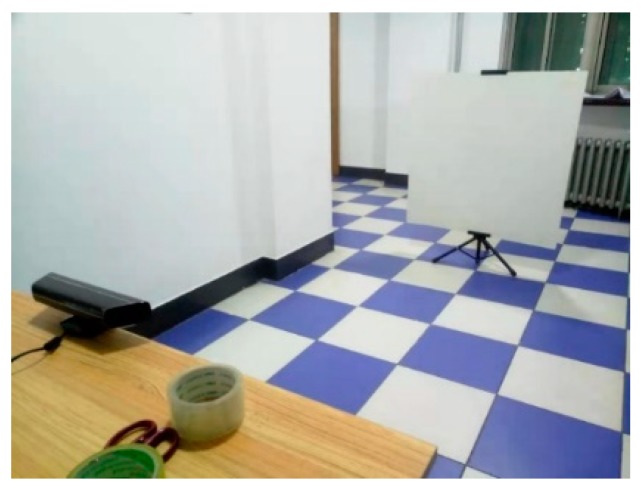
Test equipment.

The system error of the camera is estimated by calculating the difference between the average depth measurement of the Kinect V1.0 camera and the true depth. In addition to the camera, the experimental equipment also includes a flat, a 1 m×1 m white plastic plate, a rotatable tripod for holding the plastic plate, and a millimeter-precision tape measure, which are shown in Figure 4. During the experiment, the camera and the white plastic plate were placed at a fixed interval of a series of equal steps with a tape measure and the depth values at the 25 pixel points sampled from the depth map acquired from the camera were averaged; the difference between the set true depth values is used as the systematic error of the camera at this distance, where the pitch is set from 1 m to 3 m in steps of 0.2 m.

**Figure 5:**
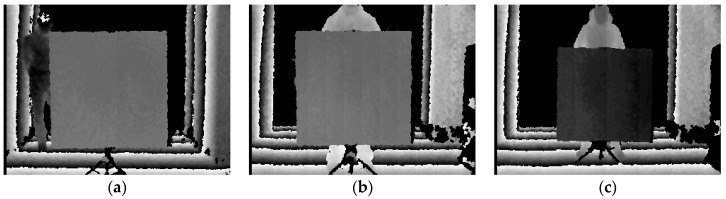
(**a**) Z = 1.8 m indoor depth map result; (**b**) Z = 1.8 m daytime outdoor depth map result; and (**c**) Z = 2.2 m night outdoor depth map result.

According to the above method, depth maps at various intervals are obtained in indoor and outdoor environments, as shown in the Figure 5.

After processing the experimental data, the average error distribution at different intervals is compared as shown.

**Figure 6:**
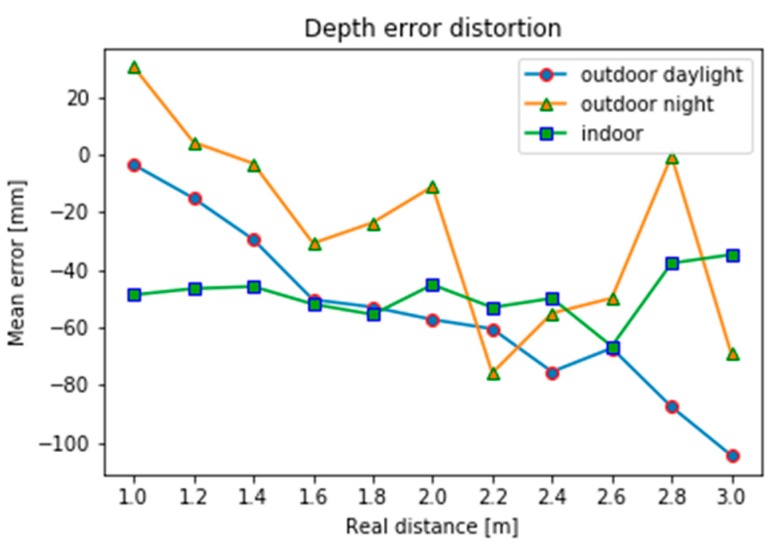
Comparison of system error with real depth under different conditions.

It can be seen from the comparison of the system errors of the camera under different conditions in Figure 6 that the outdoor system error generally satisfies with the increase of the measurement distance. In contrast, the indoor system error has no obvious law and the distribution is relatively random. After comparing indoors and outdoors, the range of indoor system errors is small, so the camera is measured indoors and the light is more dark and soft.

### 3.2. Measurement Noise Test

The depth measurement noise is calculated from the standard deviation of each pixel depth measurement. The specific experimental method is based on the previous camera system error test experiment, adding the angle of the plastic plate by rotating the tripod at each pitch to obtain the measurement result when the plastic plate is at different angles from the imaging plane of the camera. Then, it samples a number of columns of pixels corresponding to the plastic board in the depth map, and the depth value on each sampled pixel is calculated from the average of the depth measurement results of all the pixels in the column. Finally, then statistical methods are applied to the entire image, the data consisting of the deviation of the sampled pixel points is processed to obtain a deviation distribution, and then the standard deviation of the distribution is calculated as the camera depth measurement noise. In the actual experiment, the angle of the plastic plate at each pitch is set to $0^{\circ }\sim 75^{\circ }$, the step size is $15^{\circ }$, and thus there are six sets of data on each interval.

#### 3.2.1. Indoor Test

**Figure 7:**
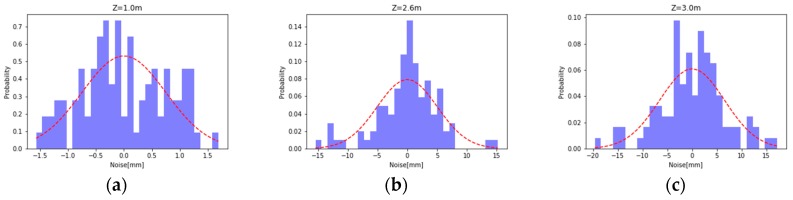
(**a**) Z = 1 m deviation distribution; (**b**) Z = 2.6 m deviation distribution; and (**c**) Z = 3 m deviation distribution.

The interval deviation statistics calculated from the sampled data on each image are obtained by interval probability statistics, and the distribution as shown in the Figure 7 is obtained. It can be seen from the figure that the distribution of the deviation basically conforms to the normal distribution with a mean of 0, and the standard of the positive distribution, i.e., the difference corresponds to the depth measurement noise we are looking for.

**Figure 8:**
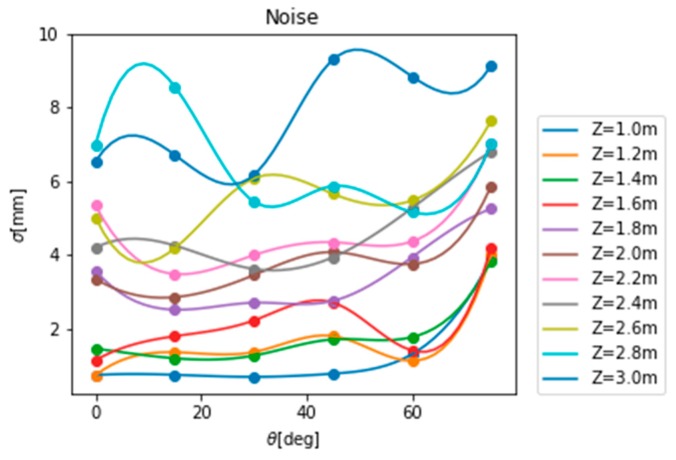
Kinect V1.0 indoor noise model.

Statistical analysis of the noise at various distances and angles gives the noise model of the Kinect V1 camera indoors, as shown in Figure 8.

It can be seen from Figure 8 that the indoor noise model of the Kinect V1 camera ranges from 0 to 10 mm, the overall noise is small, the camera performance is better, the noise model is generally consistent with the law that as the angle  θ between slope, and as the imaging plane gradually increases, the noise also gradually increases, but there is still a large randomness.

#### 3.2.2. Outdoor Test

**Figure 9:**
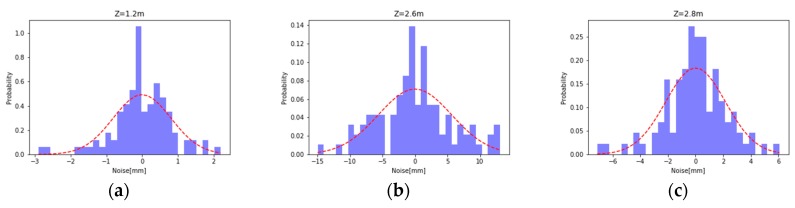
(**a**) Z = 1.2 m deviation distribution; (**b**) Z = 2.6 m deviation distribution; and (**c**) Z = 2.8 m deviation distribution.

The experimental process and data processing method are exactly the same as the indoor test, and will not be described here. The results are as follows in Figure 9.

**Figure 10:**
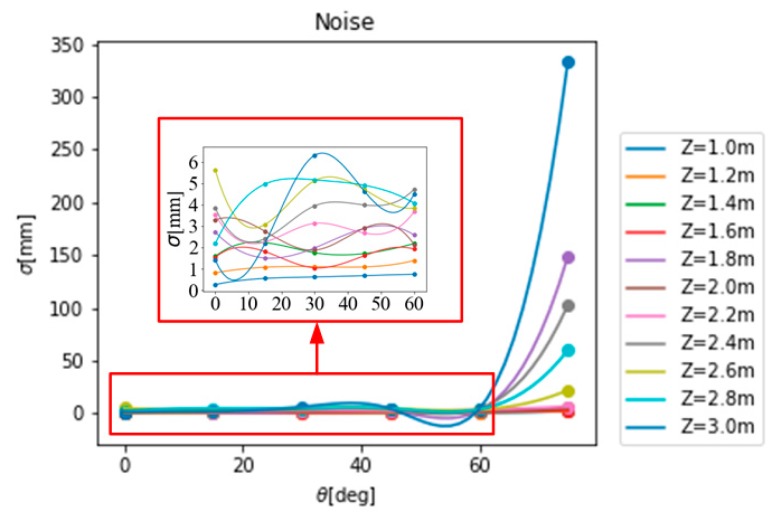
Kinect V1.0 outdoor noise model.

Statistical analysis of the noise at various distances and angles gives the noise model of the Kinect V1 camera outdoors, as shown in Figure 10.

It can be seen from Figure 10 that the outdoor noise model of the Kinect V1 camera is in the range of 0 to 7 mm within a certain angle range, and the noise influence is gradually increased after the outdoor angle is affected by the illumination and wind; thus the measurement divergence is not accurate. From the overall experiment, the noise is small, the camera performance is better, and the noise model generally is consistent with the law that as the angle  θ between slope and imaging plane gradually increases, the noise also gradually increases, but there is still a large randomness.

In summary, this kind of sensor the test uses has certain feasibility. This test combined the actual rover environment, as an example to explore the correctness of the algorithm, the follow-up will continue to carry out relevant research based on the actual rover platform.

## 4. Results and Discussion

### 4.1. Experimental Composition and Settings

This paper will test the correctness of the proposed algorithm from three aspects: (1) terrain estimation experiment based on ROS simulation environment; (2) based on Optitrack assisted indoor perception test; and (3) based on outdoor soil environment test. It should be pointed out that in order to increase the comparison level from simulation to physical verification, except for the environment differences, the unmanned running platform and the sensor are all in the same configuration, that is, the simulation environment is a high-precision simulation of the real object. Where the simulation environment will build a simulated lunar terrain based on the ROS Gazebo simulation platform and the physical test will be carried out in the indoor Optitrack auxiliary environment and the outdoor soil environment. The unmanned platform uses Jackal from Clearpath, Canada, equipped with the ROS operating environment, sensor selection for the Microsoft Kinect V1.0 depth camera, AFT601D triaxial vibrotactile sensor, and single-axis gyroscope. In addition, the terrain reconstruction of this paper will be based on the Universal Grid Map Library described by Bloesch [31].

### 4.2. Simulation Test

**Figure 11:**
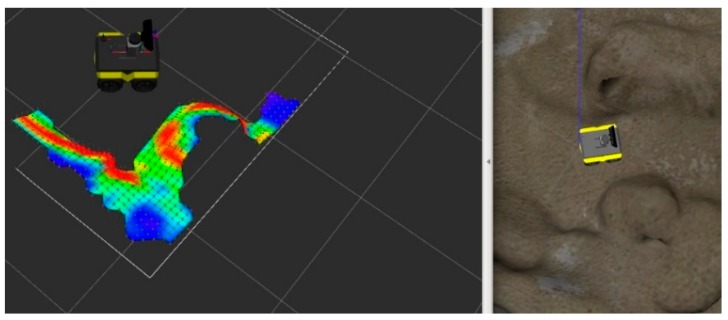
Robot operating system (ROS) simulation environment indication and mapping results.

This section is based on the simulated moon surface simulation environment built by ROS Gazebo, as shown in Figure 11. The right picture shows the running display, and the left picture shows the partial terrain display reconstructed from the running process. It can be seen from the second part that the reconstruction accuracy of the terrain is improved by estimating the height and covariance of each detection point, where, on the base of estimation, 3σ distribution estimates to better judge the rationality of terrain estimation.

**Figure 12:**
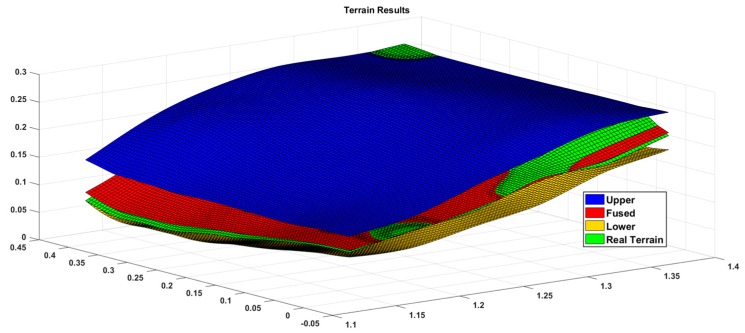
Terrain mapping results.

**Figure 13:**
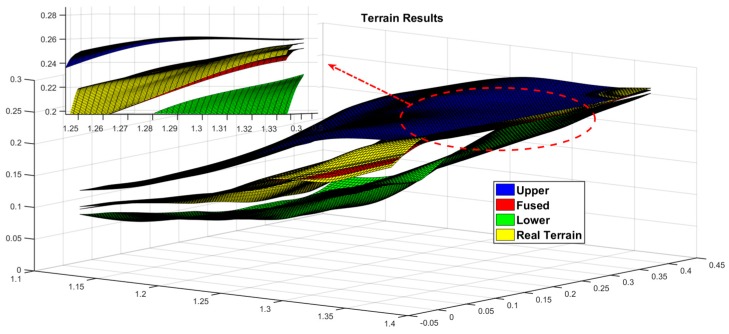
Details of the intersection of the terrain.

Figure 12 shows the estimated results of the terrain, where the blue and yellow surfaces are the 3σ upper and lower boundaries of terrain estimates, the red surface is the estimated terrain result, the green surface is the actual value of the terrain, most of the regions satisfy the estimated value, and the real value in the reasonable boundary region, And, in combination with Figure 13, it can be clearly seen that the estimated value and the true value are almost coincidence, because the test area is an uphill trend, the estimated boundary value at the later stage is significantly reduced, and it can be seen that the estimation accuracy in the flat area is higher than that in the sloped area.

**Figure 14:**
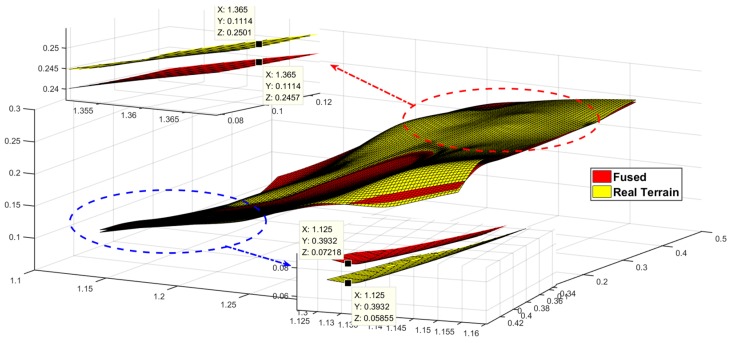
Comparative analysis of the estimated terrain and real terrain.

In order to compare the error between the estimated value of the algorithm and the actual terrain value, as shown in Figure 14, the details of the red and blue regions are compared. From the red elliptical region, the maximum height estimation error is 0.44 cm when the x and y values are the same; from the blue elliptical area, the maximum height estimation error is 1.363 cm when the x and y values are the same. This error satisfies the accuracy requirements in actual operation compared to the diameter of the mobile platform wheel.

**Figure 15:**
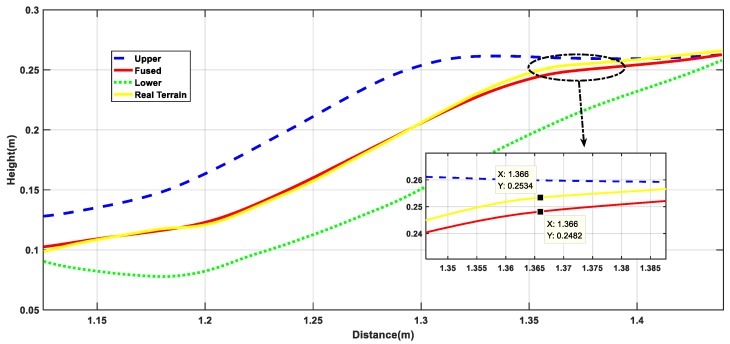
Terrain estimation profile results.

**Figure 16:**
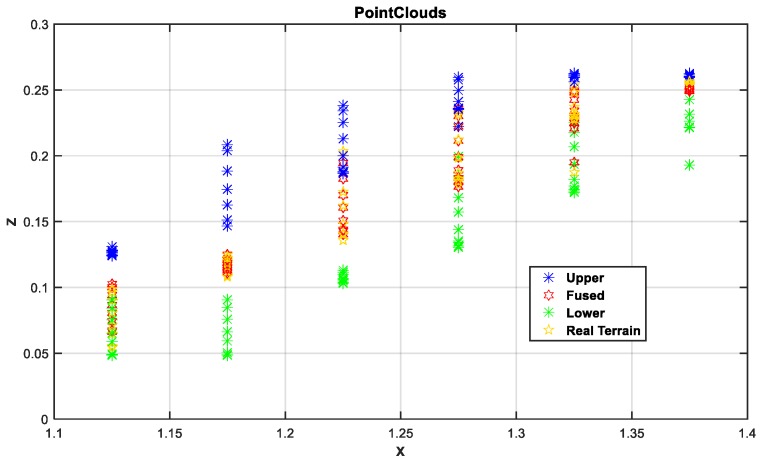
Terrain estimation section point cloud results.

Next, based on the results of the above terrain surface, the estimation accuracy of the algorithm for the irregular terrain is analyzed from the height estimation profile. It can be seen from Figure 15 that the estimated value represented by the red curve and the true value represented by the yellow curve almost coincide in the operation process, they are completely within the reasonable upper and lower boundaries, and the height deviation is 0.52 cm from the partial enlargement diagram, indicating that the rationality of algorithm to irregular terrain estimation. From the 3σ distribution area on the terrain, it can be seen that the distribution boundary is narrower than the undulating part in the relatively flat part of the initial and the end, which is also consistent with the features of high accuracy of flat terrain estimation and large estimation deviation of the undulating terrain. At the same times, the distribution of point cloud maps in Figure 16 can also verify the above points. In general, from the reconstruction results of the simulation environment algorithm, the accuracy is still ideal, but after all, compared with the real environment, there are still gaps in the environmental noise, operational deviation, and the uncertainty of the wheel interaction. Therefore, the next section will perform performance comparison tests based on the actual platform and sensing unit in the actual environment.

### 4.3. Optitrack-Based Indoor Test

**Figure 17:**
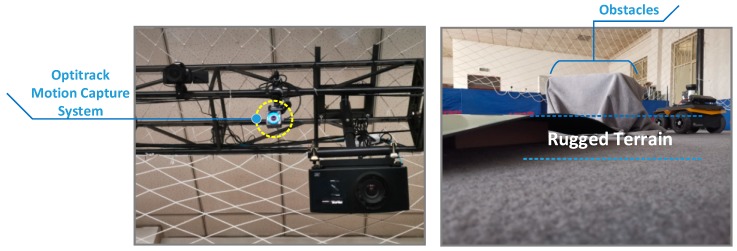
The indoor verification environment.

**Figure 18:**
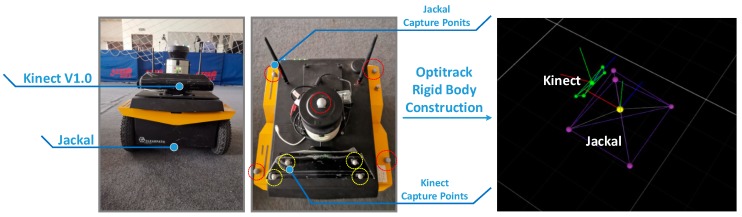
Experimental platform and capture of rigid body construction in Optitrack.

In this section, the simulated terrain is built in the Optitrack experimental environment. As shown in Figure 17, the site contains obstacles and simulated undulating terrain. At the same time, the capture points are arranged on the unmanned vehicle platform and the sensor, so as to construct the tracking rigid body in the Optitrack environment. As shown in Figure 18, the experimental platform capture is given.

**Figure 19:**
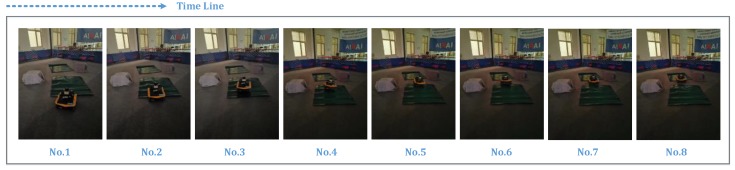
Experiment at different times.

**Figure 20:**
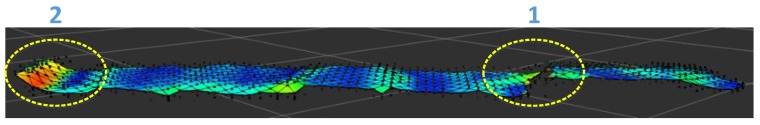
Terrain mapping result.

The experimental process is shown in Figure 19, which shows the progress of the journey at different times. Figure 20 shows the reconstruction results of the above terrain. Here, in order to improve the calculation efficiency, the sampling range of the vision sensor is limited. From the results, it can be seen that the terrain fluctuation trend is basically the same as the set terrain. At position 1, corresponding to the actual terrain slope where the change is large, the sensor is unable to detect the slope in the blind zone due to the tendency of the car to tilt upward. Therefore, there will be a missing phenomenon in the terrain. In the subsequent analysis, the change between the sampling points trend fitting achieves a full-join estimation of the terrain. At position 2, the actual terrain is a green carpet, which is clearly detected from the experimental results as it is bent to a certain curvature at the end of the carpet.

**Figure 21:**
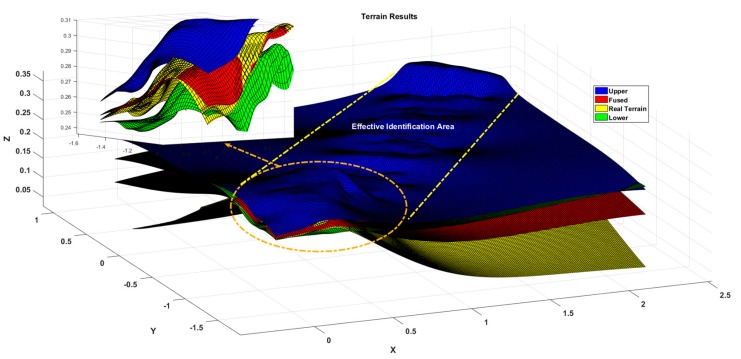
Comparative analysis of terrain mapping results.

Figure 21 shows the experimental results of the test terrain, where the red surface is the estimate of the terrain and the blue and green surfaces represent the terrain estimates and the 3σ upper and lower distributed boundaries, respectively; the yellow surface is the actual topographic value. According to the limitation of the measurement range of the sensor, the range of the effective estimation area is given by the yellow dotted line in the figure, and the detailed display of the larger deviation is given. From the small figure, we can see that the estimated and actual values of the terrain are within the upper and lower boundaries and the upper and lower boundary ranges are less than 5 cm.

**Figure 22:**
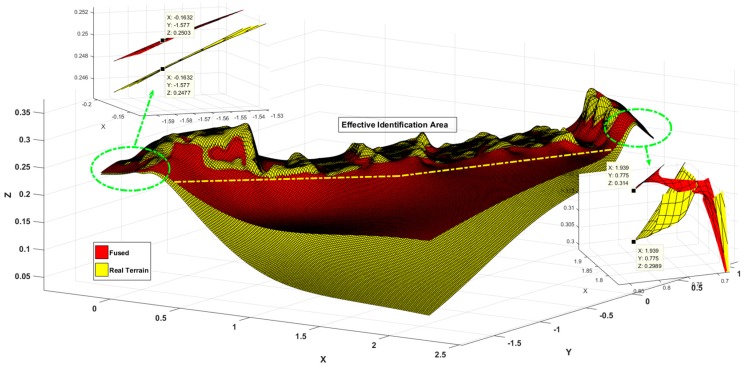
Comparative analysis of terrain estimates and real values.

At the same time, combined with Figure 22, the error between the estimated value and the true value of the terrain can be compared. For effective terrain recognition area, a detailed map of the terrain is given and the same x and y points are selected for height contrast. The comparison of values, as shown by the image on the left, is at a relative flat terrain error of 0.26 cm, as shown by the image on the right, with a height deviation of 1.51 cm at the end. The process deviation is also basically within 2 cm of the deviation.

**Figure 23:**
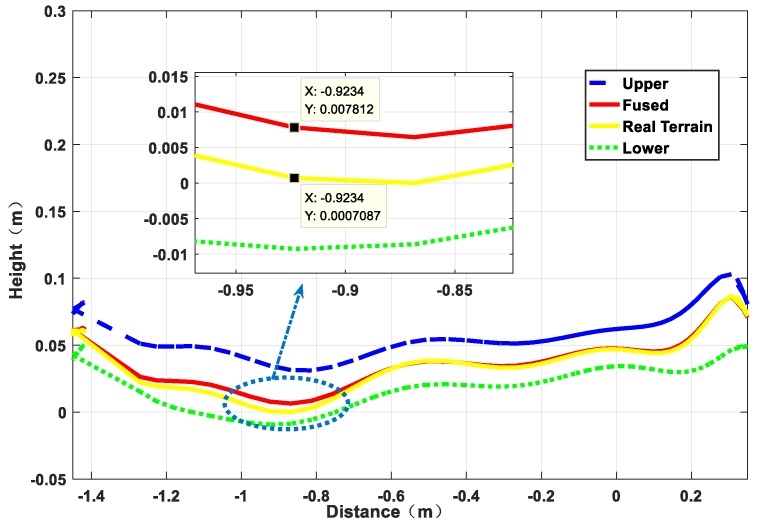
Terrain mapping results profile.

**Figure 24:**
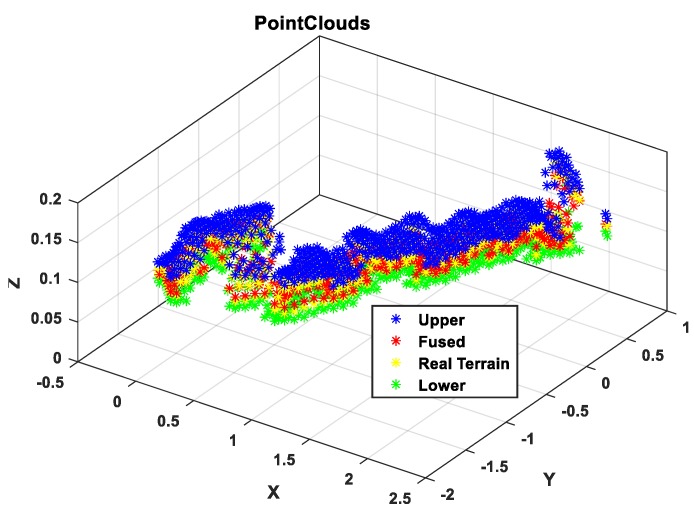
Terrain mapping 3D point clouds.

Figure 23 is an analysis of the terrain profile. Because this experiment is not based on a fixed straight line, it completely follows the 3D actual motion process. Therefore, the terrain profile corresponding to the intermediate measurement value is selected for analysis. From the large image, the estimated value deviates from the true value and the largest deviation part is in the range of the blue dotted line. As can be seen from the detail picture, the height deviation corresponding to the same x value is in the range of 0.8 cm. Because the selected reference section is where the camera sampling is concentrated and averaged, the measurement accuracy is relatively high. While, from the curve trend, the 3σ distribution range is relatively wider where the terrain changes more, which is also consistent with the feature of high accuracy of flat terrain estimation and large deviation of undulating terrain estimation. Combined with the global point cloud information shown in Figure 24, it can be analyzed that the overall estimation effect of the terrain is close to the actual one. Compared with the diameter of the platform wheel, the error magnitude is completely in accordance with the actual operation process.

### 4.4. Outdoor Test

**Figure 25:**
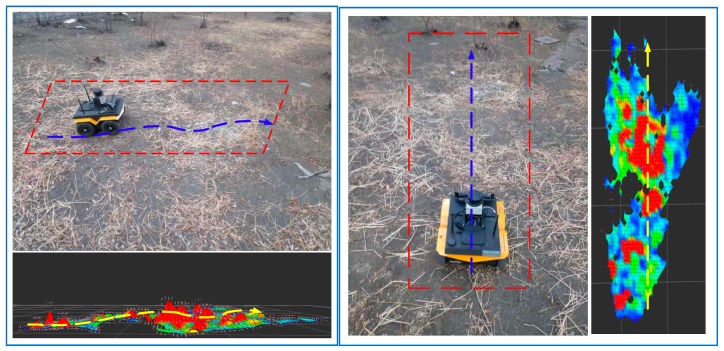
Outdoor test results.

After completing the above test, we have a quantitative understanding of the estimation accuracy of the algorithm, but, after all, compared with the actual operating environment, both the terrain fluctuation and environmental impact are ideal. Therefore, this section will be based on the simulated ground land environment for testing; an analogy to the situation when the celestial body is operating outside the Earth. Figure 25 shows the experimental environment and the reconstructed topographic results. It can be seen from the figure that the actual operating environment is complicated, except for the soil and weeds, which brings a lot of interference to the actual estimation. It can be seen from the running comparison that the perceived terrain trend is roughly consistent with the trend of travel.

**Figure 26:**
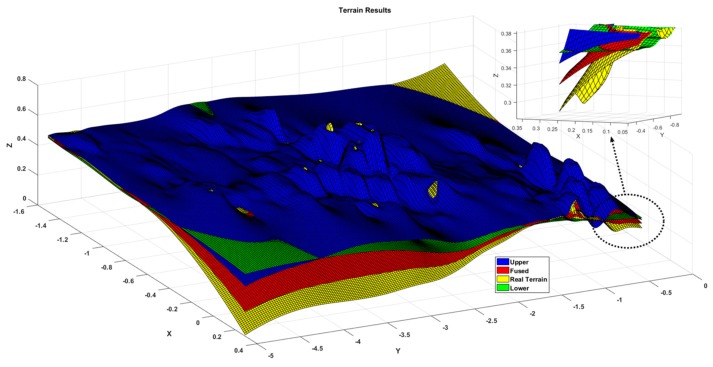
Comparative analysis of terrain mapping results.

**Figure 27:**
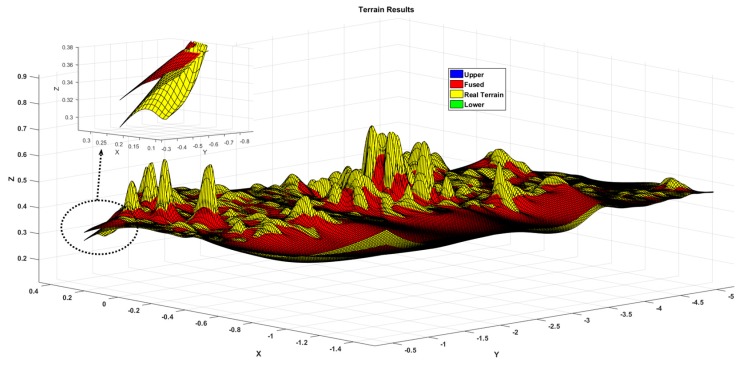
Comparative analysis of terrain estimates and real values.

**Figure 28:**
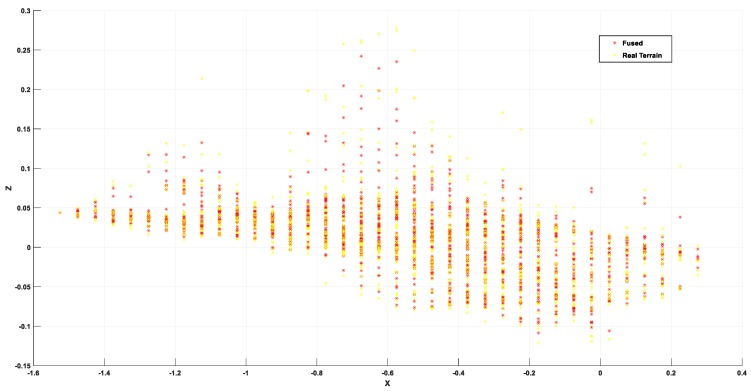
Point clouds results.

Figure 26 shows the test results in an outdoor land weed environment. Since the terrain fluctuations cannot be accurately measured and the experimental distance is relatively close, the odometer measurement value is approximated as the true value. The terrain reconstruction results are shown in Figure 27. From the effective detection area in the middle part, it can be seen that the terrain fluctuation is large and the detail range is close to 9 cm, which shows the intersection of the distribution surfaces. This explains why the outdoor operation environment interference is quite large and why the estimated values of the algorithm are easily biased by sensor measurements, platform motion, and sensor-to-platform solid-state deviations. From Figure 28, it can be concluded that the maximum deviation of the two positions in the height direction is close to 8 cm, but most of them are within 3 cm deviation, which has a certain relationship with the uncertainty of the environment; the approximate real terrain and the actual terrain also have certain difference. From the subsequent path planning results, it can be seen that the accuracy of terrain reconstruction can meet the actual needs in practical applications, so it can be estimated that the terrain estimation deviation is within 6.5 cm.

**Figure 29:**
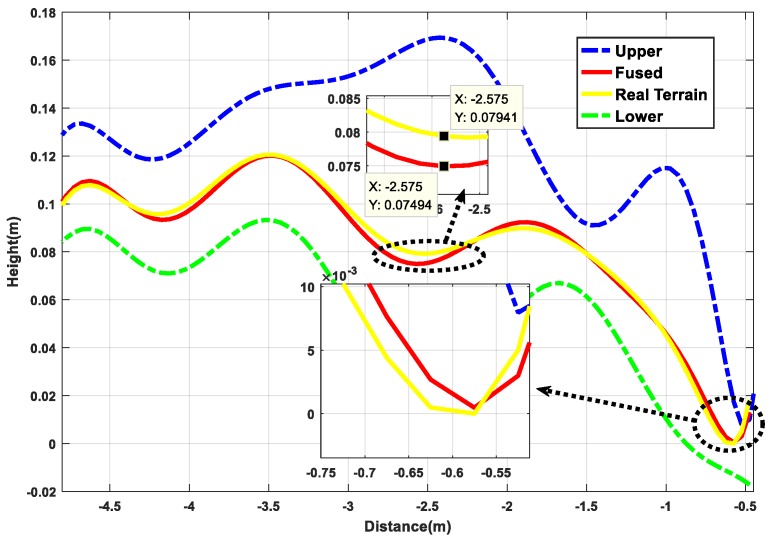
Terrain mapping results profile.

Figure 29 shows the results of the terrain profile of the running process; compared to the previous two tests, 3σ distribution interval is obviously larger. When the operation stops due to the inertia of the platform, the unevenness of the terrain, and the inevitable slight flutter of the sensor and the platform in the platform motion environment, the estimation uncertainty will be caused. The intersection between the surfaces appears, which indicates that the external influence of the actual operation process is large.

## 5. Conclusions

In this paper, based on the terrain reconstruction requirements of planetary rover process, a vibration/gyro-coupled terrain estimation method considering the influence of uncertainty is proposed, which takes into account the uncertainty of sensor measurement and the uncertainty of platform motion. Based on the ROS Gazebo simulation platform, indoor Optitrack environment, and outdoor land environment, the results show that the terrain estimation algorithm proposed in this paper has good estimation accuracy in the simulation environment and indoor environment. The simulation environment accuracy is 1 cm within the centimeter and the estimated accuracy in the indoor environment is less than 2 cm, but due to the influence of the environment, the estimated deviation is large in the uneven outdoor soil environment. Although the actual terrain cannot be accurately obtained, it can be concluded that the estimated error should be less than the height of the platform based on the operation situation of the unmanned platform carrier, that is, less than 6 cm, which has a certain practical value compared with the diameter of the hub of 20 cm. Based on the research results of this paper, the proposed sensor configuration and reconstruction algorithm has centimeter-level reconstruction capability, but it still needs to be deeply studied in the global high-precision estimation optimization and semantic terrain construction in large-scale environment, providing reliable environmental awareness protection for planetary inspection.

## Figures and Tables

**Table 1:** Microsoft Kinect V1.0 parameter list.

Parameter		Kinect V1.0
Color	Resolution	640 × 480
	FPS	30 fps
Depth	Resolution	320 × 240
	FPS	30 fps
Examination Range		0.8–4.0 m
Angle	Horizontal	57°
	Vertical	43°
